# Comparison of different approaches of percutaneous vertebroplasty in the treatment of osteoporotic spinal compression fractures and analysis of influencing factors of re-fracture

**DOI:** 10.12669/pjms.39.1.7069

**Published:** 2023

**Authors:** Dan Bu, Xuejun He

**Affiliations:** 1 Dan Bu, Department of Orthopedics, Hunan Provincial People’s Hospital, The First-Affiliated Hospital of Hunan Normal University, Changsha 410100, Hunan Province P.R. China; 2 Xuejun He, Department of Orthopedics, Hunan Provincial People’s Hospital, The First-Affiliated Hospital of Hunan Normal University, Changsha 410100, Hunan Province P.R. China

**Keywords:** Osteoporotic vertebral compression fracture, Percutaneous vertebroplasty, Access mode, Risk factors

## Abstract

**Objective:**

To compare the functional and radiological outcome of different approaches of percutaneous vertebroplasty (PVP) in the treatment of osteoporotic vertebral compression fractures (OVCF), and to analyze the factors affecting postoperative re-fracture in patients with OVCF.

**Methods:**

Medical data of 76 patients with OVCF who underwent PVP in our hospital from January 2021 to December 2021 were analyzed retrospectively. Based on the different intraoperative approaches, patients were divided into Unilateral-group (n=36) and Bilateral-group (n=40). The perioperative indexes, clinical efficacy, and spinal nerve function of the two groups were compared. Logistic regression analysis was used to determine the risk factors of postoperative re-fracture in patients with OVCF. The functional outcome was assessed with Oswestry disability index (ODI), American Spinal Injury Association (ASIA) nerve function classification and pain with Visual analogue scale (VAS). The radiological outcome was assessed by noting change of anterior vertebral height and change of kyphosis Cobb angle.

**Results:**

The amount of intraoperative bleeding, the number of X-ray irradiation and the volume of injected bone cement in the Unilateral-group were lower, and the operation time was shorter than Bilateral-group (all *P*<0.05). One week after the operation, the anterior height of the vertebral body was higher, the Cobb angle of kyphosis was lower, the VAS score was higher, and the ASIA grade was lower in the Unilateral-group compared to the Bilateral-group (*P*<0.05). Logistic regression analysis showed that the age, bone mineral density, volume of bone cement injection and PD were risk factors of postoperative re-fracture in patients with OVCF.

**Conclusion:**

Unilateral PVP treatment of OVCF has the advantages of less intraoperative bleeding, less X-ray irradiation and shorter operation time. At the same time, bilateral PVP is associated with improved bone cement dispersion, and the effect of improving patients’ pain is better than that in the Unilateral PVP. Postoperative risk of re-fracture in OVCF patients correlated with age, bone mineral density, amount of bone cement injection and pedicle diameter.

## INTRODUCTION

Percutaneous vertebroplasty (PVP) is an effective method for the clinical treatment of OVCF. Using a puncture needle to penetrate the vertebral body from the pedicle and inject bone cement can enhance the strength and structural stability of the vertebral body and reduce bone pain.^1^,^2^ PVP can reduce the risk of complications such as lower extremity deep venous thrombosis and prolapse pneumonia caused by long-term bed rest with conventional conservative treatment.^3^ However, since the degree of osteoporosis of patients varies, the clinical efficacy of different approaches of PVP is different. Studies show that bilateral PVP, which is considered a preferred OVCF treatment, is associated with increased exposure to radiation.^4^

Unilateral PVP approach has shown to be beneficial in reducing operation and radiation exposure time, lowering the risk of cement leakage and rate of possible complications.^4^,^5^ However, safety and efficacy of unilateral approach is still unclear. Additionally, the reports from the clinical practice suggest that some OVCF patients can have repeated new fractures after the operation, which prolongs the recovery time of patients. Identifying the factors that affect postoperative re-fracture rate is, therefore, important to improve the prognosis of OVCF patients.^6^ The choice of PVP approach is still controversial, and the literature on postoperative re fracture is limited, so this study retrospectively compared the clinical data of patients with unilateral and bilateral PVP approaches, in order to explore a better approach and the factors that affect the re fracture rate of patients with OVCF after surgery.

## METHODS

Medical records of 76 OVCF patients treated with PVP in our hospital from January 2021 to December 2021 were analyzed retrospectively; Including 18 males and 58 females; the age was 54-93 years old, with an average of 71.00±8.98 years old; Fracture location: 12 cases in thoracic segment, 46 cases in thoracolumbar segment, and 18 cases in lumbar segment; 26 cases of postoperative re-fracture; Patients were divided into Unilateral-group (n=36) and Bilateral-group (n=40) according to different intraoperative approaches.

### Ethical Approval:

This study was approved by the medical ethics committee of our hospital (Approval No.: 2021-190; Date: 2021-08-20).

### Inclusion criteria:

- OVCF diagnosed by imaging examination combined with clinical manifestations, and the time from fracture to admission ≤ 12 hours;
- Bone mineral density (BMD) t value at admission ≤ -2.5 SD; Successful PVP operation;
- CT three-dimensional reconstruction performed after the operation;
- Complete follow-up data for three months or more after the discharge available.

### Exclusion criteria:

- Congenital deformity of the spine;
- Patients with stroke, spinal cord nerve injury and Alzheimer’s disease;
- Bone cement allergy or incomplete clinical data.

For the PVP procedure, the patient was placed in a prone position. The area of interest was confirmed by fluoroscopy, and the vertebral body of spinal fracture was located using the C-arm X-ray machine. The projection of the pedicle of the injured vertebral body was marked, and intravenous general anesthesia or local infiltration anesthesia in the direction of the pedicle was performed, followed by routine disinfection and towel laying.

For the unilateral conventional transpedicular approach, the position of 1-cm at the outer edge of the body surface projection of the pedicle of the injured vertebra at two or 10 points was taken as the puncture point. The position of the puncture needle was adjusted according to the actual pedicle of the patient under X-ray fluoroscopy, and a working sleeve was placed when the tip of the puncture needle reached 1/3 behind the injured vertebra.

For the bilateral conventional transpedicular approach, two points on the right and 11 points on the left of the outer edge of the body surface projection of the pedicle of the injured vertebra were taken as the puncture point. The position of the puncture needle was adjusted according to the actual pedicle of the patient under X-ray fluoroscopy, and a working sleeve was placed when the tip of the puncture needle reached 1/3 behind the injured vertebra.

After confirming that the working cannula was placed, the prepared high viscosity bone cement (Teknimed, France) was slowly pushed into the injured vertebral body through the working cannula under the X-ray machine fluoroscopy. During this procedure, the fluoroscopy image was closely monitored. When the bone cement dispersion approached the posterior edge of the injured vertebral body, cement dispersion was paused for 30 seconds, the outer sleeve was pulled out, and fluoroscopy was performed again after ten minutes to confirm that the bone cement in the injured vertebral body is intact. Medical gauze was used to compress the puncture point after the operation, and sterile application was applied after confirming that there was no bleeding. After operation, zoledronic acid sodium (Novartis, Switzerland), alendronate sodium (Hangzhou MSD Pharmaceutical Co., Ltd., China), ibuprofen tablets (Anhui Renhe Pharmaceutical Co., Ltd., China), and calcitonin (Novartis, Switzerland) were given according to the actual situation of patients to promote blood circulation and reduce swelling.

The following basic data of patients and related indexes before and after the operation were collected.

### Perioperative indicators:

intraoperative bleeding, X-ray irradiation times, bone cement injection, bone cement leakage and operation time.

### Clinical efficacy:

evaluated from four aspects: the change of anterior vertebral height, the change of kyphosis Cobb angle, pain relief and the change of Oswestry disability index (ODI);^7^an online survey on CLBP was conducted. HRQoL was measured with two specific questionnaires, i.e. Oswestry Disability Index (ODI Visual analogue scale (VAS) was used to evaluate the pain of patients. The full score of VAS was 10, and the degree of pain was positively correlated with the score.^8^

Spinal cord nerve function was evaluated by the American Spinal Injury Association (ASIA) nerve function classification^9^ before the operation, and one week, one month and three months after the operation. ASIA is divided into I~V levels according to the impairment of spinal cord function. The higher the level, the better the spinal cord function. Logistic regression analysis was used to determine the risk factors of postoperative re-fracture in patients with OVCF.

Prognosis evaluation was made by comparing the clinical data of patients with postoperative re-fracture and no fracture, including gender, age, bone mineral density, number of vertebral fractures, PVP surgical indicators, postoperative imaging indicators kyphosis Cobb angle, length of the pedicle trail (LPT), pedicle diameter (PH), pedicle trail (PD).

### Statistical analysis:

SPSS 22.0 statistical software was used to analyze the research data. The measurement data were described by (*χ̄*±*S*), and t-test was used; The counting data are described in n (%), use *χ^2^* inspection, Logistic regression analysis was used to determine the risk factors of postoperative re-fracture in patients with OVCF, and the results were presented as odds ratios (or) with 95% confidence interval (CI). P<0.05 was considered statistically significant.

## RESULTS

The unilateral-group consisted of eight males and 28 females; The average age was 73.38±10.07 years; BMD index 3.20±0.42; fracture location: six cases of thoracic segment, 20 cases of thoracolumbar segment, ten cases of lumbar segment; 12 cases of postoperative re-fracture, including two thoracic segments, six thoracolumbar segments and four lumbar segments;. Bilateral-group: 10 males and 30 females; The average age was 76.08±10.10 years old; BMD index was 3.26±0.59; Fracture location: six cases of thoracic segment, 26 cases of thoracolumbar segment, eight cases of lumbar segment; 14 cases of postoperative re-fracture, including two thoracic segments, nine thoracolumbar segments and three lumbar segments; there was no significant difference in general data between the two groups (*P*>0.05). As summarized in Table-I, the amount of intraoperative bleeding, the number of X-ray irradiation and the amount of injected bone cement in the Unilateral-group were significantly lower than in the Bilateral-group, and the operation time was shorter than that in the Bilateral-group (*P*<0.05).

**Table-I:** Comparison of perioperative indexes between the two groups [n(%),*χ̅*±*S*].

Group	n	Intraoperative bleeding (ml)	X-ray exposure times (Times)	Bone cement injection volume (ml)	Cement leakage	Operation time (minutes)
Unilateral-group	36	31.55±7.42	12.22±3.69	3.19±1.03	8 (22.22)	36.89±6.44
Bilateral-group	40	40.35±5.77	23.45±5.34	5.67±1.28	7 (17.50)	53.95±11.63
t/χ^2^	-	5.794	10.743	9.178	0.267	8.006
P	-	<0.001	<0.001	<0.001	0.606	<0.001

One week after the operation, the height of anterior edge of the vertebral body in both groups was higher than that before the operation, Table-II and significantly higher in the Unilateral-group compared to the Bilateral-group (*P*<0.05). The kyphosis Cobb angle, VAS score and ODI in the two groups were lower than those before operation. The kyphosis Cobb angle in the Unilateral-group was significantly lower than that in the Bilateral-group (*P*<0.05), while the VAS score in the Unilateral-group was higher than that in the Bilateral-group (*P*<0.05); there was no significant difference between the two groups in each item at one month and three months after operation (P>0.05) (Table-II). Postoperative ASIA score in both groups was higher than that before the operation (*P*<0.05). ASIA score in Bilateral-group after the surgery was higher than that in Unilateral-group (*P*<0.05) Table-III.

**Table II:** Comparison of clinical efficacy between the two groups (*χ̅*±*S*).

Item		Time			Unilateral-group(n=36)	Bilateral-group(n=40)			t/χ^2^	P
Height of anterior edge of vertebral body (mm)		Preoperative			29.69±3.18	30.25±3.14			0.764	0.447
		One week after operation			34.50±3.45^a^	32.67±3.32^a^			2.347	0.022
		One month after operation			35.33±3.27^a^	34.07±3.54^a^			1.602	0.113
		Three month after operation			35.50±3.30^a^	34.8±3.44^a^			0.839	0.404
Kyphosis Cobb angle (°)		Preoperative			42.86±4.25	43.25±4.34			0.394	0.695
		One week after operation			21.19±2.24^a^	24.62±3.09^a^			5.482	<0.001
		One month after operation			19.67±2.19^a^	20.17±2.98^a^			-0.840	0.403
		Three month after operation			17.97±2.33^a^	1815±3.02^a^			-0.285	0.777
VAS score		Preoperative			7.16±1.05	7.37±0.95			0.905	0.369
		One week after operation			5.25±0.96^a^	4.47±1.03^a^			3.467	0.001
		One month after operation			2.89±0.71^a^	2.60±0.98^a^			1.481	0.143
		Three month after operation			2.03±0.61^a^	1.98±0.77^a^			0.330	0.743
ODI (%)		Preoperative			74.19±6.87	75.25±6.21			0.703	0.484
		One week after operation			32.61±4.33^a^	31.90±4.08^a^			0.736	0.464
		One month after operation			28.03±4.37^a^	27.57±3.84^a^			0.481	0.632
		Three month after operation			25.53±4.70^a^	24.40±3.86^a^			1.147	0.255

*Group*	*n*	*Height of anterior edge of vertebral body (mm)*			*Kyphosis Cobb angle (°)*		*VAS score*		*ODI (%)*	

		*Preoperative*	*One week after operation*		*Preoperative*	*One week after operation*	*Preoperative*	*One week after operation*	*Preoperative*	*One week after operation*

Unilateral-group	36	29.69±3.18	34.50±3.45^a^		42.86±4.25	21.19±2.24^a^	7.16±1.05	5.25±0.96^a^	74.19±6.87	32.61±4.33^a^
Bilateral-group	40	30.25±3.14	32.67±3.32^a^		43.25±4.34	24.62±3.09^a^	7.37±0.95	4.47±1.03^a^	75.25±6.21	31.90±4.08^a^
t/χ^2^	-	0.764	2.347		0.394	5.482	0.905	3.467	0.703	0.736
P	-	0.447	0.022		0.695	<0.001	0.369	0.001	0.484	0.464

***Note:***

a indicates that compared with preoperative, *P* < 0.05

**Table-III:** Comparison of ASIA score between unilateral and Bilateral-groups (*χ̅*±*S*).

Group	n	ASIA classification score			

		Preoperative	One week after operation	One month after operation	Three months after operation
Unilateral-group	36	1.58±0.60	3.05±0.58^a^	4.22±0.63^ab^	4.67±0.53^abc^
Bilateral-group	40	1.47±0.50	3.47±0.55^a^	4.52±0.55^ab^	4.87±0.33^abc^
t		0.851	3.206	2.215	2.010
P		0.398	0.002	0.030	0.049

***Note:***

a represents the preoperative comparison with the same group, *P*<0.05;

^b^ indicates comparison of preoperative values with values one week after the operation in the same group, *P*<0.05;

^c^ indicates comparison of preoperative values with values one month after the operation in the same group, *P*<0.05.

As summarized in Table-IV, compared with patients without postoperative fracture, patients with postoperative re-fracture were ≥ 80 years, had lower bone mineral density, lower bone cement injection volume, and smaller pedicle diameter (PD). The difference between the groups was statistically significant (*P*<0.05). Logistic regression analysis showed that age ≥ 80 years old, low bone mineral density, lower volume of injected bone cement and low PD were the risk factors of postoperative re-fracture in patients with OVCF. Table-V

**Table-IV:** Comparison of clinical data between patients with postoperative re fracture and patients without fracture [n (%),*χ̅*±*S*]

Factors	Postoperative re-fracture (n=26)	No fracture after operation (n=50)	t/χ^2^	P
Gender (Female)	16 (61.53)	38 (76.00)	1.739	0.187
Age (≥80 years)	16 (61.53)	7 (14.00)	18.317	<0.001
Bone mineral density (T-value)	-3.54±0.49	-3.07±0.45	4.229	<0.001
Number of vertebral fractures (PCS)	1.57±0.50	1.38±0.49	1.646	0.104
Thoracic segment fracture	2(7.69)	10(20.00)		
Thoracolumbar segment fracture	16(61.54)	30(60.00)		
Lumbar segment fracture	8(30.77)	10(20.00)		
Approach mode (Unilateral)	14 (53.85)	22 (44.00)	0.665	0.415
Bone cement injection volume (ml)	3.61±1.52	4.96±1.62	3.488	0.001
Bone cement leakage (yes)	6 (23.08)	9 (18.00)	0.278	0.598
Cobb angle of kyphosis one week after operation (°)	22.76±3.30	23.12±3.18	0.450	0.654
LPT (mm)	21.96±3.52	20.86±3.00	1.428	0.157
PH (mm)	10.57±1.88	11.06±2.44	0.956	0.343
PD (mm)	5.68±1.16	7.02±1.36	4.292	<0.001

LPT- length of the pedicle trail; PH-pedicle diameter, PD-pedicle trail.

**Table-V:** Risk factors for postoperative refracture of OVCF by logistic regression analysis.

Factors	β	SE	Waldx^2^	P	OR	95%CI
Age ≥ 80 years	1.735	0.805	4.653	0.031	0.176	0.036~0.853
Low bone mineral density	3.203	1.011	10.035	0.002	0.041	0.006~0.295
Less bone cement injection	0.684	0.300	5.201	0.023	1.982	1.101~3.567
PD small	1.137	0.365	9.687	0.002	3.116	1.523~6.375

PD-pedicle trail.

## DISCUSSION

Percutaneous Vertebroplasty (PVP) is of the optimal method for the clinical treatment of OVCF and can provide sufficient stabilization of the fracture and a quick pain relief. During the procedure, bone cement is injected into the injured vertebral body after percutaneous puncture through the pedicle or extra pedicle. This helps to enhance the strength and change the stability of the vertebral body. Zhang G et al.^10^ showed that PVP with high viscosity bone cement for OVCF can reduce the incidence of bone cement leakage, which is helpful to further reduce pain and improve bone metabolism. At present, the choice of PVP approach is still controversial for elderly OVCF patients who can tolerate surgery.^11^

By comparing the application effects of different approaches of PVP in OVCF, our study showed that patients that receive unilateral PVP had less intraoperative bleeding, and required shorter X-ray irradiation times and lower volume of the injected bone cement than those treated with bilateral PVP. Unilateral PVP was associated with shorter operation time, and the height of anterior edge of vertebral body one week after operation was higher than that of Bilateral-group patients. Our results are in agreement with the study of Zhang et al^12^ that indicated that the operation risk of unilateral PVP approach is smaller than that of bilateral PVP, and speculated that the operation time and X-ray irradiation time of bilateral approach are longer. As cement leakage is one of the most frequent complications of PVP, the intraoperative injection of bone cement on one side may increase the possibility of leakage on the other side, thus potentially increasing the risk, associated with the bilateral approach.

Recent studies show that the biomechanical changes after spinal fracture may gradually lead to local kyphosis.^13^ The stress of the adjacent vertebral body on the injured vertebral body increases, while the pressure load that the vertebral body can bear decreases, resulting in pain and other associated symptoms. The results of our study demonstrated that the Cobb angle of kyphosis in the bilateral PVP group was greater than that of the Unilateral-group one week after the operation, and the VAS score was lower than that in the Unilateral-group. These results suggest that the bilateral approach PVP is more efficient in restoring the stability of the injured vertebral body and alleviating the short-term pain after the operation.

Additionally, the results of our study also showed that the ASIA classification level of the Bilateral-group was higher than that of the Unilateral-group one week after the operation, indicating that the bilateral approach PVP is beneficial for the recovery of spinal cord function in OVCF patients. This may be due to the balanced distribution of bone cement on both sides of the injured vertebral body during the bilateral PVP procedure. The injected cement can then fully diffuse into the bone trabecular structure, improve the stiffness of the injured vertebral body, further balance and strengthen the biomechanical properties of the vertebral body, and help to maintain the biological force line of the spine.^14^ The puncture angle of unilateral approach PVP is large, which increases the possibility of the spinal cord and nerve injury by the puncture needle entering the spinal canal. This may explain lower ASIA classification scores of the Unilateral-group compared to the Bilateral-group one week after the operation.^15^ There is a need to further explore the factors affecting the recovery of spinal cord function in patients with OVCF.

With the development of minimally invasive spinal surgery, there have been increasing reports of postoperatively fractured vertebral body or adjacent vertebral body re-fracture in OVCF patients after PVP.^16^ Previous studies show that the incidence of post-PVP re-fracture is about 11%, while the incidence of postoperative re-fracture in our study was about 34%. The reason for this large difference may be related to the small sampling size in our study. Logistic regression analysis confirmed that age, bone mineral density, bone cement injection and pedicle trail (PD) were the risk factors of postoperative refracture in patients with OVCF. With the increase of age, the intake of calcium gradually decreases, while the outflow of calcium gradually increases.

The decrease of calcium content in blood induces the rise of secondary thyroid hormone, and finally reduces the content of calcium ions deposited in bone and increases the risk of fracture.^17^ Bone mineral density is related to the internal structure of the bone tissue. After the decrease in bone mineral density, the stress borne by the body cannot be effectively distributed in the vertebral body, resulting in repeated collapse of the surgical vertebral body or adjacent vertebral body.^18^ PVP mainly uses the viscous effect of bone cement after full diffusion and solidification in the fracture space of the injured vertebra to fix the bone trabecula and to alleviate the pain. Insufficient injection of bone cement may easily cause uneven diffusion of the bone cement in the fractured vertebral body. That may lead to unstable internal structure, which changes the ratio of PD and the anterior and posterior height of the vertebral body, and thus increases the risk of postoperative re-fracture.^19^

### Limitations:

This is a retrospective design and shorter follow up study, and sample size is small. Therefore, there is a need for further larger studies, clinical trials and studies with longer follow-up times to evaluate the prognosis of patient.

## CONCLUSION

Unilateral approach PVP has the advantages of less intraoperative bleeding, less X-ray irradiation and shorter operation time. Bilateral PVP has the advantages of short-term pain relief and good recovery of spinal nerve function. The incidence of postoperative re-fracture is related to the patient’s age, bone mineral density, the volume of injected bone cement and the pedicle trail.

### Authors’ contributions:

**DB** conceived and designed the study. **DB and XH** collected the data and performed the analysis. **DB** was involved in the writing of the manuscript and is responsible for the integrity of the study.

All authors have read and approved the final manuscript.
